# Fresher with flavour: young women smokers’ constructions and experiences of menthol capsule cigarettes and regular cigarettes

**DOI:** 10.1186/s12905-021-01297-2

**Published:** 2021-04-16

**Authors:** E. Gilbert, A. Ewald

**Affiliations:** School of Psychology, Western Sydney University, Locked Bag 1797, Penrith, NSW 2751 Australia

**Keywords:** Smoking, Young women, Menthol, Gender, Flavour capsule cigarettes

## Abstract

**Background:**

Flavour capsule cigarettes are one of the fastest growing segments of the tobacco market, and there is evidence that Australian young people are increasingly using menthol flavoured capsule cigarettes. This qualitative research examines how young women construct and experience menthol flavour capsule cigarettes as part of their smoking practices, and explores the perceived differences between menthol capsule cigarettes and regular cigarettes. Semi-structured face-to-face in-depth interviews were conducted with 41 Australian young women smokers, using a constructivist grounded theory approach.

**Results:**

Findings reveal that the perceived fresh and improved taste of menthol and the ability to customise the smoking process positively contributed to young women’s experiences of smoking menthol capsule cigarettes. In particular, menthol capsule flavour cigarettes were constructed by the young women as “fresh”, “light” and “minty”, and “popping” the menthol capsule allowed the young women to personalise their smoking experience.

**Conclusion:**

These results indicate that specific public health campaigns and legislation should be developed to counter the powerfully alluring effects and the innovative appeal of menthol capsule cigarettes.

**Supplementary Information:**

The online version contains supplementary material available at 10.1186/s12905-021-01297-2.

## Introduction

Australia is a global leader in the implementation of tobacco control policies [1]; pioneering the world’s first plain tobacco packaging legislation in 2012 [2, 3]. This legislation has been endorsed by the World Health Organization [4] and is a measure that has since been implemented by other countries across the globe (e.g. [5–7]). The core aims of plain packaging are to reduce cigarette appeal, increase the salience of health warnings, and reduce perceptions of harm [1], with recent studies providing support for the effectiveness of plain packaging [2–11]. However, there is a well-documented history of the tobacco industry innovating products in the face of bans and legislation [2, 12, 13], and the industry’s response to plain packaging has been no different [14, 15]. Since plain packaging, tobacco companies have continued to develop novel pack sizes, extra-long and slim cigarettes, super-value brands [16], innovative filters, and flavour capsule cigarettes [17]. The recent innovations in filters are a crucial strategy used by the industry to maintain brand differentiation and promotion [18], with flavour capsule cigarettes amongst the most important filter innovations in the tobacco sector [19]. In Australia, flavour capsule cigarettes were introduced during the lead up to plain packaging legislation [20] and have not since been prohibited. In fact, flavour capsules are one of the fastest growing segments of the tobacco market globally [21, 22] as well as in Australia [23] and are not included in plain packaging lesislation. Preference for capsule cigarettes grew significantly from 1% to approximately 3% in Australia in the two years after plain packaging implementation, with capsules particularly popular with young people [23]. One of the most popular flavour capsules are menthol capsules [23] which are regular cigarettes that can be given a menthol flavour by crushing a ball in the filter of the cigarette which contains a menthol liquid [20].

There is a wealth of well documented problematic public health concerns surrounding the use of menthol flavour cigarettes (see [24–28]), however, the relative novelty of menthol flavour capsule cigarettes has meant that there is a dearth of research examining their use. Flavour capsule cigarette use is an area of research priority for the World Health Organisation [29], especially since menthol capsules are specifically targeted to, and used by, young people [30, 31], with older people more likely to position capsules as a gimmick [32]. While age is consistently identified as a factor shaping capsule use, there is limited research on other identity factors that are associated with capsule use; specifically, gender. There is a critical need for research in this area as menthol flavour capsules appeal to young women [19, 33], and the tobacco industry has a rich history of feminising menthol cigarettes [17, 34, 35] as well as marketing certain cigarettes as essential to women’s gender performance [36, 37]. Previous research has also highlighted differential preferences in relation to women’s and men’s smoking, including that females prefer menthol flavour more than their male counterparts, and that taste characteristics of cigarettes are of more importance to women than to men [34]. The few studies that have examined young women’s use of menthol capsule cigarettes reveal a number of problematic health and gendered insights [31]. For example, in their focus group study, Moodie et al. [32] found that young women ranked menthol cigarettes as more pleasant tasting, less smelly, and less harmful that regular cigarettes. Capsules were also seen as ‘cool’ and ‘funky’, and especially appealed to young women because they were seen as being able to be shared amongst friends. Young women are also particularly likely to report that capsules may encourage non-smokers to experiment, smokers to consume more, and to discourage quit attempts [38].

While a few qualitative studies have addressed menthol capsule use [17, 32, 39], much of what we know about menthol capsule cigarettes stems either from reviews of tobacco industry documents [40, 41], or from quantitative and experimental research designs; with a focus on measuring trends or consumer perceptions of, and behavioural intentions around, menthol capsules [42–45]. The primacy of quantitative and experimental research in this area means that we are lacking the subjective experience of menthol capsule smoking, and in-depth insight into how young women’s experience of menthol flavour capsule cigarette use is linked to gender. To add to the growing body of research on menthol flavour capsule cigarettes, in this qualitative interview study we examine how young women who are current smokers construct and experience menthol flavour capsule cigarettes as part of their smoking practices, and explore the perceived differences between menthol capsule cigarettes and regular cigarettes.

## Method

### Participants

Forty-one female university students volunteered to take part in the research. Participants ranged in age from 18 to 37 years, with an average age of 22 years. The cultural identities of the young women were diverse, with the participants self-identifying their cultural background as either: Middle Eastern (N = 14), European (N = 9), Australian (N = 6), Asian (N = 3), Oceanic (N = 2), African (N = 2) or mix of cultures (N = 5). Thirty-three participants self-identified as heterosexual, 4 as bi-sexual, 3 as lesbian, and 1 participant chose not to disclose her sexual identity. Australian smoking rates are particularly high in lower socio-economic populations [46], with this study including participants primarily from these populations. Using the Australian Bureau of Statistics’ Socio-economic Indexes for Areas (SEIFA) [47], all the young women resided in low socio-economic geographical locations. The majority were first generation in family to attend University, with the majority reporting that their parents also smoked cigarettes, and were employed in lower skilled occupations [48].

The age at which participants began smoking ranged from 11 to 20 years (mean 17 years). The usual number of cigarettes smoked per day ranged from 0 to 25, with the majority (N = 29) of women smoking 5 cigarettes or less per day, 8 participants smoking 6–10 cigarettes per day, 3 participants smoking between 11–25 cigarettes per day, and 2 participants describing their daily smoking as ‘variable’. The young women self-identified as either regular or social smokers, with 16 participants reporting smoking between 0–10 days out of the past 30 days, 9 smoking between 11–20 days out of the past 30 days, 15 participants smoking 21–30 days over the past 30 days, while 1 participant was ‘unsure’ how much she smoked during the past 30 days. In addition, 18 reported smoking ‘as much on my own as with others’ whilst 23 reported ‘mostly smoking with others’. The majority (N = 23) reported smoking menthol flavour capsule cigarettes, and only one participant reported that she smoked menthol cigarettes without a capsule. It should be noted that within Australia menthol or mint is the only permitted flavouring to be added to cigarettes, and all of the participant’s who smoked a capsule flavoured cigarette smoked a menthol capsule cigarette. Of the remaining 18 participants, 14 young women reported smoking regular cigarettes (unflavoured and without a capsule), 3 smoked either slim sticks, roll your own cigarettes, and 1 participant was unsure of the cigarette type she smoked. Of the 23 menthol flavour capsule smokers, Winfield Optimum Crush Blue was the most common brand smoked. Of the 18 young women who did not smoke flavour capsule cigarettes, Winfield was also the most common brand smoked.

### Procedure

Ethical approval for the study was received from Western Sydney University Human Research Ethics Committee (H13025), and all the research design protocol for involving humans was in accordance to guidelines of the National Statement of Ethical Conduct in Human Research. Participants were recruited through an online Australian University research recruitment platform in which University students are allocated course credit in exchange for research participation. The participants were purposively selected on the basis that they identified as women aged between 18 and 40, and that they were ‘current’ smokers. Current smoking status was defined as smoking cigarettes daily, weekly, or less often than weekly [46]. Sampling occurred until theoretical saturation was reached with no new or relevant data emerging [49]. Prior to participating, participants were asked to read an information form detailing the nature of the research, sign informed consent acknowledging their voluntary participation, and complete a short background questionnaire including demographic questions as well as questions about smoking behaviour, type(s) of cigarette smoked, brand smoked, and typical context of smoking (Additional file 1).

Semi-structured face-to-face in-depth interviews were then conducted with women smokers to explore current insights and experiences with smoking, the feelings attached to smoking cigarettes, thoughts on plain packaging, cigarette types and brands smoked; and while exploring the women’s experiences of menthol capsule cigarettes was not initially a focus of this research, as the interviews progressed it was clear that preferences for capsule or regular cigarettes was an important issue to explore. The interview schedule can be found in the appendix. Author one conducted all the interviews, which were one-off, usually lasted for forty-five minutes to one hour, and took place from May to December 2019. All of the interviews were audio- recorded and transcribed verbatim. The excerpts that appear in this paper have been edited only to the extent that a few irrelevant sentences and words have been replaced with ellipses, and excessive uses of colloquialisms, such as ‘like’ and ‘um’ have been removed. Square brackets indicate certain undercurrents in the interview, such as [pause] or [laughter]. When sentences trail off without further elaboration, … is used as an indicator. To preserve anonymity, all of the young women have been given pseudonyms.

### Analysis

A Constructivist Grounded theory approach was used to generate a theory around the context of the shared phenomenon of cigarette smoking and involved the systematic integration of the data collection and data analysis stages [50, 51]. As the interview process progressed and the research was refined, ‘theoretical sampling’ was employed, which is sampling on the basis of the representativeness of concepts that have theoretical relevance to the evolving theory [51]. This meant that the research was inductive, where the concepts and categories came from the data, rather than being deductive or informed by existing preconceptions about smoking. As an example, the initial iteration of the interview schedule focused primarily on women’s feelings about cigarette smoking and what they thought about plain packaging. However, as the interviews progressed, and data was collected and simultaneously analysed, the interview schedule (Additional file 2) was constantly redeveloped and refined to reflect the participant’s accounts—with ‘flavour capsule cigarettes’ a key issue discussed by all the young women.

For the analysis, each interview transcript was manually coded, and, to attain a high level of abstraction, three stages of Grounded Theory coding were employed. The data were first subject to ‘open coding’, involving a close reading of each transcript to identify ‘first-order concepts’. In-vivo labels/concepts were attached to pieces of the data and this began the initial process of data inference. A series of coded concepts were created around the young women’s understandings of cigarette smoking, and cigarette type and brands. Some of these included ‘the tobacco taste’, the tobacco smell’, ‘inhaling the smoke’, ‘the burn’, ‘the filters’, ‘refreshing’, ‘minty’, ‘menthol flavour’, ‘crushing/popping the capsule’. After creating first-order concepts, ‘axial coding’ was employed to develop ‘categories’ that subsumed many of the first-order concepts. During the axial stage of coding, the relationships between categories were explored, and new connections and recoding was carried out. To give some examples, ‘the tobacco taste’, ‘the tobacco smell’, ‘inhaling the smoke’, and ‘the burn’ were joined to create the category; ‘Physical sensations associated with smoking’. The categories ‘the filters’, ‘refreshing’, ‘minty’, ‘menthol flavour’ and ‘crushing/popping the capsule’ were joined to create the category; ‘Menthol Capsule Flavour Cigarettes are ‘Fresh’, ‘Light’, and ‘Minty’. As the analysis proceeded, and in the final stage of ‘selective’ coding, the axial codes entitled: ‘Menthol Capsule Flavour Cigarettes are ‘Fresh’, ‘Light’, and ‘Minty’; ‘Popping’ the Menthol Capsule and Personalising the Smoking Experience’ and ‘Regular Cigarettes have more flavour and substance’ were developed which linked all of the associated concepts and categories. The core category which resulted was ‘Freshness with Flavour’, allowing for the dataset to be theorised.

## Results

This qualitative research aimed broadly to explore the meanings young women’s attach to cigarette smoking and plain packaging. However, during the research process, the women’s constructions and experiences of menthol capsule flavour cigarettes was found to be the dominant and overarching theme. Within this overarching theme, menthol capsule flavour cigarettes were constructed as “fresh”, “light” and “minty”, and were seen as improving the smoking experience.

### Menthol capsule flavour cigarettes are ‘fresh’, ‘light’, and ‘minty’

Most young women gave accounts that menthol capsules improved the taste of cigarettes, with participants describing the menthol flavour as ‘fresh’, ‘refreshing’, ‘like a blast of mint’ (Grace), and ‘not as bitter’ (Emika) as regular cigarettes. As Meredith said, ‘It’s almost like a breath of fresh air. They just seem a bit cleaner, a bit fresher’. For Emika, menthol capsules ‘kind of feels like healthier in a way because it's more refreshing’ and ‘it's more like an air-con in your mouth’. For many, the menthol capsules also made the cigarettes feel ‘lighter’, ‘milder’, less ‘harsh on your throat’ (Sarah)’, ‘not as strong’ as regular cigarettes, and allowed them to avoid ‘that disgusting taste in my mouth’ (Tenika). As Cassandra described;

I don't like harsh cigarettes. They (Winfield Optimum Crush) feel very, very light. That's why I like them. Especially if you pop them at the start, they're extremely light. Enjoyable and lighter, yeah.

Some young women explained that when compared with menthol flavour capsule cigarettes, regular cigarettes are ‘just dirty’, ‘dirty tasting’, ‘horrendous’, and ‘look yuck’. In fact, some participants, like Meredith provided accounts that ‘if I can’t get my menthol cigarettes, I won’t smoke’. Unlike regular cigarettes which are described by many of the young women as ‘tasting like a chemical’ (Claudia), menthol flavour capsule cigarettes are like smoking a sweet or breath mint—‘they don’t even taste like a cigarette. They taste like you're just smoking a minty” (Adeline). As Aylin said;

There's like this little ball, you pop it. Some people literally take it out and just taste it. Like it pops in your mouth and it's just like mint.

The mint flavour of the menthol capsule masked the taste of tobacco, with participants like Nala saying; ‘you don't really taste the tobacco as much’, which makes the cigarette seem fresher and ‘cleaner’. For Meredith, the feeling of cleanness extended beyond the taste of the menthol capsule cigarette to the smell also;

I have this weird thing in my head that maybe—it’s more subconscious—that I won’t smell of cigarettes either, because if I can’t taste the dirtiness, I can’t smell the dirtiness. They just seem a bit cleaner, a bit fresher.

### ‘Popping’ the menthol capsule and personalising the smoking experience

Most young women reported crushing the capsule to release the menthol flavour all of the time. Capsules were crushed at the beginning of smoking—‘before the first puff’ (Rashida) or ‘before I light it’ (Claudia), ‘after a few drags’ (Emika), ‘towards the end’ (Heidi). ‘Popping’ the capsule reportedly allowed the young women to interact with the cigarette and personalise their smoking experience, as Chloe said, ‘Like you'll get half-way through and then it tastes weird and then you remember to pop it’. Lena said ‘I'll have it normal and then towards the end I'll pop it’. When asked why she crushes at the end of smoking, Lena replied ‘to get the mint fresh feeling’. Similarly, Giorga explained how crushing the menthol capsule halfway through smoking allows her to have ‘the best of both worlds’;

I smoke half plain, and then I crush it. I know people will crush it straight up, but I like to have the best of both worlds (…) I need to have it even. So, I need to smoke it straight up, normal, and then when I get to a certain point, I need to pop it.

When asked what the cigarette would taste like if the menthol capsule was not crushed, many said ‘it would taste like an actual cigarette’ (Sarah) or ‘taste like shit’ (Giorga). In contrast, crushing the menthol capsule reportedly results in ‘a minty feeling in my mouth (Grace), ‘a better taste’ (Serena), because ‘it doesn’t have that disgusting taste in my mouth, and also, it’s not as harsh as just smoking it on its own’ (Tenika)*.* When discussing what they liked about crushing the capsule many participants said it was because as Aylin explained, ‘it pops and it tastes good’ and ‘whenever it starts getting bad, I pop it’.

### Regular cigarettes have more flavour and substance than menthol flavour capsule cigarettes

Almost all of the eighteen young women who did not smoke flavour capsule cigarettes reported that they ‘can’t stand menthols’ (Stephanie) or the ‘minty kind of taste’ (Inessa). For Amisha, menthol capsules make the cigarette taste ‘plasticky’ and ‘artificial’, while Stephanie described menthol capsules as ‘like somebody ate like a whole bunch of mints and then threw up in your mouth’. Instead, the flavour of regular cigarettes was seen by many as ‘a lot nicer’ (Kyah) and ‘smoother’ (Sabeen) than menthol capsule cigarettes, with many preferring the ‘full bodied flavour and texture’ (Daniela) and ‘the pure tobacco taste’ (Amisha) of regular cigarettes. For example, when talking about Marlboro Red, Daniela said;

I like the burn of the cigarette, Okay. I like the drawing into my lungs and exhaling it's like taking a deep breath, but like, there's more substance to it.

Chiara also talked about ‘the heat that you get’ from inhaling regular cigarettes ‘and that kind of rush’ makes ‘everything become clearer than it did’, and ‘makes me feel present. It makes me feel in the moment’. Similarly, Stephanie, who smoked Rothman Black cigarettes said; ‘cause when I'm stressed it kind of like burns but it like centres me (…) So it's like it grounds me ‘cause I have a lot of anxiety’.

## Discussion

The majority of the young women smoked menthol capsule cigarettes and discussed the increasing popularity of menthol capsules amongst young women, adding to growing evidence that capsule cigarettes are ‘attractive’ and ‘popular’ amongst young people [52]. The higher use of capsules amongst younger than older people, is a pattern across countries, including Australia [21, 23, 32]. Menthol capsule smokers gave accounts that the flavour of menthol capsules improved the taste of regular cigarettes, confirming other recent research that capsules are perceived as ‘tasting better’ than regular cigarettes [53]. In particular, previous research has revealed that female menthol smokers were significantly more likely than males to emphasise the importance of taste, smell and sensation in relation to their attitudes and beliefs about smoking [54]. Regular cigarettes were positioned as dirty, harsh, and disgusting by capsule smokers who saw capsule cigarettes as fresher, lighter, milder, cleaner, and less harsh than regular cigarettes, also supporting other research in this area [32, 39, 55]. A proportion of young women in the study smoked regular cigarettes, and reported that when compared with menthol capsule cigarettes, they preferred the flavour and tobacco taste of regular cigarettes. For these young women, menthol capsule cigarettes were positioned as ‘plasticky’ artificial, or too minty, and were experienced as detracting from the substance and ‘full-bodied’ texture of regular cigarettes. In larger more representative study samples, it has been found that non-daily smokers are more likely to prefer flavoured menthol capsules than daily smokers [56], however, we found no major differences between the number of social and regular smokers who preferred menthol capsule cigarettes over regular cigarettes—potentially due to our smaller sample size. Indeed, the majority of women smokers in our study do not reflect the ‘average’ Australian smoker, who would normally smoke more than five cigarettes per day; indicating that perhaps flavor capsule smokers do not smoke as much as regular cigarette smokers. Indeed, other research has shown that capsules are ‘starter products’ [57], and a larger more representative study is required to examine this issue in more detail; comparing smoking patterns and consumption across daily and social smokers of regular cigarettes and capsules.

We also found that menthol capsule cigarettes were likened in taste by many of the young women to a sweet or breath mint; with the menthol capsule masking the flavour and taste of tobacco to the extent that these cigarettes were seen less as ‘real cigarettes’ and more as confectionary. Whilst some existing research has detailed cooperative arrangements between the manufacturers of tobacco and those of candy cigarettes [58], our finding that menthol capsules are positioned as akin to confectionary warrants further investigation, as it is a potential strategy used to recruit children and young people into smoking. This is also particularly important because the tobacco industry has a history of strategically marketing menthol cigarettes as having taste benefits [25], with menthol and other capsule flavourings more recently used by the tobacco industry to mask the harshness of tobacco and improve the palatability of the product [59]. At the same time, decades of evidence show us that the tobacco industry has recruited young women into using particular types of cigarettes, in particular, mentholated cigarettes. For example, internal tobacco industry documents have noted that: ‘the smoking behaviour of female smokers is different from that of male smokers’; ‘women are more highly motivated to smoke than men and find it harder to quit smoking’ [60], and tobacco marketing campaigns ‘harness’ and ‘exploit social forces’ to develop classes of cigarettes targeted to women who either adhere to or resist dominant social constructions of femininity [61]. Indeed, the ‘success’ of Virginia Slims was due in large part to the fact that: “It’s a male cigarette company saying, in effect, ‘woman, we put you on a pedestal. We think you are terrific, you’re fabulous, you’re great. We love you, and we want you to have this cigarette we made for you” [62]. Historically, menthol cigarettes were seen as a progressive way by competitors to lure women away from Virginia Slims, and were targeted at young and culturally diverse women cultural identities and promoted as ‘refreshing’ [63]; a significant contribution to the construction of a discourse surrounding menthol cigarettes. As the findings of our study show, such a discourse continues to dominate for young women smokers of menthol capsule cigarettes, with menthol capsules not only seen as refreshing, but also associated with other traditionally feminine cigarette characteristics such as ‘light’, ‘mild’, and ‘clean’ [64].

In this study we also found that almost all the young women smokers of menthol capsule cigarettes reported crushing the capsule all of the time, with crushing occurring at various stages of the smoking process, including at the beginning, after a few puffs, half-way through, or at the end of smoking, confirming other research [23]. For these young women, capsules allowed them to interact with the cigarette and personalise their smoking experience, findings that are also consistent with other research showing that capsule cigarettes are technologically appealing [29], that capsules provide ‘customisation’ and ‘consumer interaction’ with cigarettes, and that young people enjoy ‘clicking the capsule’ to change from a regular to menthol flavour [38]. Whilst the findings of our study and other recent research alert us to the innovative appeal of capsule cigarettes, further research is needed to examine the potential individual and collective social allure of the mass personalisation of capsule cigarettes.

Several limitations of this study require addressing; related largely to sampling. We assessed only women’s constructions and experiences of smoking practices and capsule use. Evidence shows the use of capsules across genders, with young men also attracted to the allure of menthol capsules. A systematic comparison of men and women’s use of capsules is required to clearly identify the gendered aspects of capsule use and smoking more generally. Also, as this was a qualitative study, our sample was relatively small, with participants recruited from a single University, and the women were largely light smokers. Future research is required to address the issue of capsule smoking in a larger more representative sample if we are to adequately address the lack of research on capsules, and attend to what may be a significant failing in global public health. Even in our small sample, there are significant implications for policy and practice resulting from these findings, including informing legislation and public health campaigns around smoking, and in providing a fuller understanding of young women’s attraction to menthol capsule cigarettes.

In conclusion, the findings from this study lend support to research urging the banning of menthol capsule cigarettes in an effort to improve public health [65, 66], as well as provide evidence for the increased legislation of plain tobacco products to include innovative filters in Australia and internationally. This is a particularly important recommendation in the light of emerging international research showing support for menthol bans amongst menthol smokers from across vulnerable populations [67, 68], and evidence that a menthol ban would motivate menthol smokers to quit; reducing the tobacco-related disease and death [69]. At the same time, it is critically important to evaluate bans on flavoured cigarettes, such as the ban that has been introduced across the European Union, as well as the United Kingdom, since May 2020. In addition, we suggest that specific public health campaigns should be developed to counter the powerfully alluring effects of menthol capsule cigarette types and brands, especially amongst young women.

## Supplementary Information

**Additional file 1.** Demographic survey for young women smokers.

**Additional file 2.** Interview schedule for young women smokers.

## Data Availability

The datasets used and/or analysed during the current study are available from the corresponding author on reasonable request.
