# Supplementary material for: Fresher with flavour: young women smokers’ constructions and experiences of menthol capsule cigarettes and regular cigarettes
Source: BMC Womens Health. 2021 Apr 16;21:155. doi: 10.1186/s12905-021-01297-2 (PMC8051088; doi:10.1186/s12905-021-01297-2)
Supplement: Supplementary file 1 — Additional file 1. Demographic survey for young women smokers. [file 12905_2021_1297_MOESM1_ESM.docx]

**Demographic Survey**

**Introduction:** As part of the Young Women and Cigarette Smoking study being conducted at Western Sydney University, we are interested in your experiences of smoking. Women who smoke daily, weekly, or monthly, and who are aged between 18- 30 years, are invited to take part in this study.

The study has been approved by Western Sydney University Human Research Ethics Committee (Study ref: H13025).

**Instructions:** Taking part in the study is voluntary. The Information you provide is confidential. The survey will take about 10 minutes to complete.

**Please email your completed survey back to Emilee before you arrive for your interview.**

If you have any questions, please contact Dr Emilee Gilbert on: Tel: 02 9772 6757; Email: e.gilbert@westernsydney.edu.au

Your participation in this study is greatly appreciated.

**Questions about you**

1. What is your current age?______________________________________

2. What is your Australian postcode?________________________________

3. What is your cultural or ethnic background? (e.g., Anglo­Australian, Chinese, Hispanic)

|  |
| --- |

4. Are you currently:

Single 🞎

In a relationship 🞎

In multiple relationships 🞎

Widowed 🞎

Separated 🞎

Divorced 🞎

5. If you are in a relationship(s), how long have you been in your current relationship(s)?_______________________________________________

6. Which best describes your living situation?

I live alone  🞎

I live with my partner 🞎

I live with my parents 🞎

I live in a shared household 🞎

Other – please specific_______________

7. Which best describes your sexual identity?

Heterosexual 🞎

Lesbian 🞎

Bi­sexual 🞎

I identify as­________________

8. Do you have any children?

No 🞎

Yes 🞎

If Yes, what are their ages? ____________________________________

9. What is the highest level of education you have achieved:

🞎 Secondary School year 12 and below

🞎 Certificate I/II/III/IV

🞎 Diploma

🞎 Bachelor’s Degree/Graduate Diploma or Graduate Certificate

🞎 Postgraduate Degree

10. Are you currently studying? (e.g., School, TAFE, University, Course)

Yes 🞎 Please state where and what____________________________________

No 🞎

11. Are you in paid employment?

Yes 🞎

No 🞎

If yes, what is your occupation? _______________________________________

12. On average, how many hours of paid work do you do a week? _________________

13. On average, how many hours of unpaid/volunteer work (including domestic duties) do you do a week?______________________________

14. Please select the income bracket below that best applies to you:

🞎 $151, 000 and above

🞎 $101,000 to $150,00 per year

🞎 $81,000 to $100,00 per year

🞎 $61,000 to $80,000 per year

🞎 $41,000 to $60,000 per year

🞎 $21,000 to $40,000 per year

🞎 < $20,000 per year

🞎 I prefer not to answer

15. What kinds of activities do you enjoy doing in your free time? (e.g. tennis, movies, soccer, theatre)

 ___________________________________________________________________________________________________________________________________________________________________

16. What is the name of the last high school you went to, and what Australian State was it in?

___________________________________________________________________________

17. What is/was your parents’ occupation?

Parent 1___________________________

Parent 2___________________________

**Questions about your smoking history and smoking status**

18. What age were you when you began smoking cigarettes regularly?______________________

19. What is the usual number of cigarettes you smoke in a day?____________________________

20. On average, how many days in a week do you smoke cigarettes (0­7)?____________________

21. On average, how many of the last 30 days did you smoke cigarettes (0­30)?________________

22. What best describes your pattern of smoking?

I smoke mostly on my own 🞎

I smoke mostly with other people 🞎

I smoke as much on my own as I do with other people 🞎

Other – please state________________________________

23. What brand of cigarettes do you smoke?____________________

24. Have you always smoked that brand? Yes/No (please circle)

25. If no – what other brands have you smoked?____________________
