# Supplementary material for: Fresher with flavour: young women smokers’ constructions and experiences of menthol capsule cigarettes and regular cigarettes
Source: BMC Womens Health. 2021 Apr 16;21:155. doi: 10.1186/s12905-021-01297-2 (PMC8051088; doi:10.1186/s12905-021-01297-2)
Supplement: Supplementary file 2 — Additional file 2. Interview schedule for young women smokers. [file 12905_2021_1297_MOESM2_ESM.docx]

**Interview schedule for smokers**

**Experiences of smoking**

1. Can you tell me about when you first started smoking?

*Prompts: what was that first experience like for you; motivation, reasons for smoking, is it any different now for you when you smoke?*

1. Can you describe what it feels like for you to smoke cigarettes?

*Prompts: what does that first drag feel like?; where do you mostly smoke, with whom, and when?*

1. Do you have a particular way you like to smoke?

*Prompts: how do you hold and ash your cigarettes?; thoughts on how different people (e.g., men/ women) hold/ash their cigarettes?*

1. What do you think others think about smoking (what do you think the perception of smoking and smokers is?)

*Prompts: the media; friends; society?*

**Anti-smoking campaigns**

1. What do you think of anti-smoking messages and campaigns?

*Prompts: TV, cigarette packs, posters, and what impact does it have on your own smoking*.

1. Tell me what you think about plain packaging of cigarette packs.

*Prompts: what do you think of the colours; what about the health warnings; do you leave your pack out on display; do you remember when plain packaging was introduced; do you ever use any cigarette tins or package the cigarettes differently?*

1. Tell me more about the brand of cigarettes that you smoke?
   1. *Prompts: why that brand? Preferences for menthol capsules/regular cigarettes; thoughts on capsules.*

**Quit attempts**

1. Tell to me about your thoughts on quitting smoking.

*Prompt: do you think you’ll always smoke?*

1. Have you made any quit attempts?

*Prompts: how many, why did you attempt to quit (e.g. family pressure, health reasons), how did attempting to quit make you feel?*

**Identity**

1. If I asked you to describe yourself to me – what would you say?
